# To the Editors of the Medical and Physical Journal

**Published:** 1804-12-01

**Authors:** J. Cullurne


### *To the Editors of the Medical and Physical Journal.*

GENTLEMEN,

IN the last Number of the Medical and Chirurgical Review is an Article, stating, that when I saw the child, who was the subject of the Kensington case, on the fourth day after I had inoculated him with cow-pock virus, "I expressed myself satisfied with the appearance of the arms."

Had the author of that article taken the trouble to call upon me, I should have informed him, that I did not, nor could I consistently, speak decidedly on that point, at so early a stage, and that nothing could be more contrary to my habit, or disposition, than such hasty decision on matters of consequence.

I requested the child might be brought to my house on the Monday following, that is, on the eighth day. He was brought on that day, but not until the time appointed was considerably past, and I was unavoidably absent from home. The reason of my directing them to bring him to my house in Lower Eaton Street, Pimlico, was, because it lays considerably nearer to Kensington than the Castle Street station, where the child was inoculated. The parents, trusting too much to their own judgment, and the improperly obtruded opinions of other people, thought the child secure, and did not send him to me again, so that I did not see him afterwards till the evening before his decease. He was then sinking under the confluent small-pox. I called for a candle, and closely examined his right arm, but could trace no mark of his having had the cow-pock. I could not examine the left arm, without greatly disturbing him (which I thought improper in that advanced stage of the disease) because he lay upon his left side; however, I did not consider that as of so much consequence, because I invariably inoculate on the right arm; sometimes, though very rarely, from timidity in the subject, or other material intervening cause, I omit to inoculate in the left; therefore having found no leading mark on the right arm to determine from, and having afterwards made the most minute inquiry into all circumstances, I am of opinion, that the vaccine inoculation never took proper effect.

This being a true statement of the case, I have to request that you will do me the justice to insert it in the next Number of your Journal.

I am, &c.

